# Statistical and Criticality Analysis of the Lower Ionosphere Prior to the 30 October 2020 Samos (Greece) Earthquake (M6.9), Based on VLF Electromagnetic Propagation Data as Recorded by a New VLF/LF Receiver Installed in Athens (Greece)

**DOI:** 10.3390/e23060676

**Published:** 2021-05-27

**Authors:** Dimitrios Z. Politis, Stelios M. Potirakis, Yiannis F. Contoyiannis, Sagardweep Biswas, Sudipta Sasmal, Masashi Hayakawa

**Affiliations:** 1 Department of Electrical and Electronics Engineering, Ancient Olive Grove Campus, University of West Attica, 12244 Egaleo, Greece; d.z.politis@uniwa.gr (D.Z.P.); yiaconto@uniwa.gr (Y.F.C.); 2 Indian Centre for Space Physics, 43 Chalantika, Garia St. Road, Kolkata 700084, India; sagar94m@gmail.com (S.B.); meet2ss25@gmail.com (S.S.); 3 Hayakawa Institute of Seismo-Electromagnetics Co. Ltd. (Hi-SEM), University of Electro-Communications (UEC) Alliance Center #521, Kojimacho, Chofu, Tokyo 182-0026 1-1-1, Japan; hayakawa@hi-seismo-em.jp; 4 Advanced Wireless and Communications Research Center (AWCC), University of Electro-Communications (UEC), Chofugaoka, Chofu, Tokyo 182-8585 1-5-1, Japan

**Keywords:** 2020 Samos earthquake, seismo-electromagnetics, VLF sub-ionospheric propagation, nighttime fluctuation method, terminator time method, natural time analysis, method of critical fluctuations

## Abstract

In this work we present the statistical and criticality analysis of the very low frequency (VLF) sub-ionospheric propagation data recorded by a VLF/LF radio receiver which has recently been established at the University of West Attica in Athens (Greece). We investigate a very recent, strong (M6.9), and shallow earthquake (EQ) that occurred on 30 October 2020, very close to the northern coast of the island of Samos (Greece). We focus on the reception data from two VLF transmitters, located in Turkey and Israel, on the basis that the EQ’s epicenter was located within or very close to the 5th Fresnel zone, respectively, of the corresponding sub-ionospheric propagation path. Firstly, we employed in our study the conventional analyses known as the nighttime fluctuation method (NFM) and the terminator time method (TTM), aiming to reveal any statistical anomalies prior to the EQ’s occurrence. These analyses revealed statistical anomalies in the studied sub-ionospheric propagation paths within ~2 weeks and a few days before the EQ’s occurrence. Secondly, we performed criticality analysis using two well-established complex systems’ time series analysis methods—the natural time (NT) analysis method, and the method of critical fluctuations (MCF). The NT analysis method was applied to the VLF propagation quantities of the NFM, revealing criticality indications over a period of ~2 weeks prior to the Samos EQ, whereas MCF was applied to the raw receiver amplitude data, uncovering the time excerpts of the analyzed time series that present criticality which were closest before the Samos EQ. Interestingly, power-law indications were also found shortly after the EQ’s occurrence. However, it is shown that these do not correspond to criticality related to EQ preparation processes. Finally, it is noted that no other complex space-sourced or geophysical phenomenon that could disturb the lower ionosphere did occur during the studied time period or close after, corroborating the view that our results prior to the Samos EQ are likely related to this mainshock.

## 1. Introduction

It is widely accepted that different kinds of electromagnetic (EM) phenomena which are possibly associated with lithospheric processes have been found prior to earthquakes (EQs), while their behavior and their possible interconnection have been investigated by the scientific community for decades [1,2,3,4,5,6,7,8,9,10]. Moreover, it is also known that the ionosphere is sensitive to EQ preparation processes, and constitutes a very promising means of short-term EQ prediction [1]. Different techniques and methods have been used to reveal any kinds of statistical anomalies or any indication of specific physical meaning, by analyzing the observables related to EQ preparation processes, not only in phenomena directly related to the lithosphere, but also with phenomena in the atmosphere and the ionosphere [2,11,12]. A few hypotheses for lithosphere–atmosphere–ionosphere coupling (LAIC) have been introduced in order to clarify the involved physical mechanisms, but LAIC is still poorly understood at the moment [2,13].

Among the time series analysis methods that have been employed in seismo-EMs, two methods that can unveil the approach to criticality are the method of critical fluctuations (MCF) [14,15,16] and natural time (NT) analysis [10]. These methods have successfully been applied to pre-EQ seismic electric signals (SES), foreshock seismicity, and EM disturbances recorded by ground stations, such as ULF magnetic field data, MHz fracto-EM emissions and, VLF sub-ionospheric propagation data [9,17,18,19,20,21,22,23,24,25,26,27,28,29,30,31,32].

In this work, we investigate a very recent, strong, and shallow EQ ($M_{W}=6.9$, focal depth =12 km) that occurred in Greece on 30 October 2020, with its epicenter located in the Aegean Sea, off the coast north of the island of Samos (Greece), close to the Greece–Turkey border, hereafter referred to as the “2020 Samos EQ”. A new VLF/LF (10–47.5 kHz) radio receiver has recently been established at the University of West Attica (call sign UWA) in Athens (Greece), and has been operating in trial mode since April 2020, monitoring the lower ionosphere mainly for EQ-related anomalies. In this work we analyze the receiver data of two specific sub-ionospheric propagation paths between UWA and two VLF transmitters located in different Eastern Mediterranean countries. The first transmitter, with call sign TBB, is located in Denizköy (Turkey), and the location of the Samos EQ’s epicenter is within the 5th Fresnel zone of the corresponding propagation path, whereas the second transmitter, with call sign ISR, is located in the Negev (Israel), and the location of the Samos EQ’s epicenter is close to the border of the ISR–UWA 5th Fresnel zone, so that considering the magnitude of the specific EQ, the corresponding propagation path could possibly be disturbed [33,34], but not by much.

The amplitude data from these transmitters that were recorded by the UWA receiver were analyzed in two ways: Firstly, the amplitude data were analyzed using two statistical methods—namely, the nighttime fluctuation method (NFM), and the terminator time method (TTM) [11,35,36,37,38,39,40]. Secondly, we performed criticality analysis by means of NT analysis and the MCF. Specifically, the time series of the VLF propagation quantities resulting from the NFM analysis were analyzed using the NT analysis method in order to find any evidence of critical dynamics, following the methodology that has already been successfully applied in previous VLF sub-ionospheric propagation analysis studies [9,25]. Moreover, the unprocessed (raw) amplitude data of the receiver were analyzed using the MCF, following the methodology that has already been successfully applied to similar VLF data [9,24,30].

The analysis results obtained by means of both statistical and criticality methods revealed clear precursors over a period of approximately 2 weeks prior to the mainshock. Notably, in the application of the NT analysis method, it was found that, for some thresholds, criticality conditions continued to be satisfied even after the EQ’s occurrence (criticality starting a few days before the mainshock and continuing for a few days after). By means of the MCF, it is shown that this is a result of critical dynamics “locally surviving” soon after the mainshock. This is a result of post-EQ power laws, not accompanied by a long-range correlation, as proven by means of the autocorrelation function.

The remainder of the article is organized as follows: Section 2 provides information about the VLF/LF transmitters worldwide that are recorded by the newly installed UWA receiver, and gives some details about receiver’s operation. Section 3 presents information about the 2020 Samos EQ, and describes the analyzed data from both of the transmitters (TBB and ISR) that are used in this study. Section 4 comprises four subsections presenting brief overviews of the statistical and criticality analysis methods employed. Section 5 comprises five subsections presenting the analysis results obtained by means of the corresponding analysis methods. Finally, Section 6 presents conclusions.

## 2. The Newly Installed UWA VLF/LF Receiver

A new VLF/LF radio receiver for the study of ionospheric disturbances possibly related to EQs, as well as to other space-sourced and geophysical phenomena, has recently been installed in Athens, in the Department of Electrical and Electronics Engineering of the University of West Attica at the “Ancient Olive Grove” campus (geographic coordinates: 37.977° N, 23.673° E), and has been operating in trial mode since April 2020. We will refer to this receiver with the call sign “UWA”. The radio receiver, designed by the Hayakawa Institute of Seismo-Electromagnetics, uses a simple vertical electrical rod (monopole) antenna. The VLF sub-ionospheric propagation data (amplitude and phase) for all of the monitored paths, between UWA and multiple transmitters worldwide (see Table 1) are recorded with a sampling period of 1 s, synchronized by a GPS receiver, and finally stored to a personal computer in the form of text files with timestamps, using the UltraMSK software. Note that the choice of a 1 s sampling period enables us to detect disturbances of the lower ionosphere possibly related to EQs, which excludes fast changes (duration < 1 s) in the signal that are attributed to phenomena out of the scope of our research, such as lightning discharges [1].

**Figure 1:**
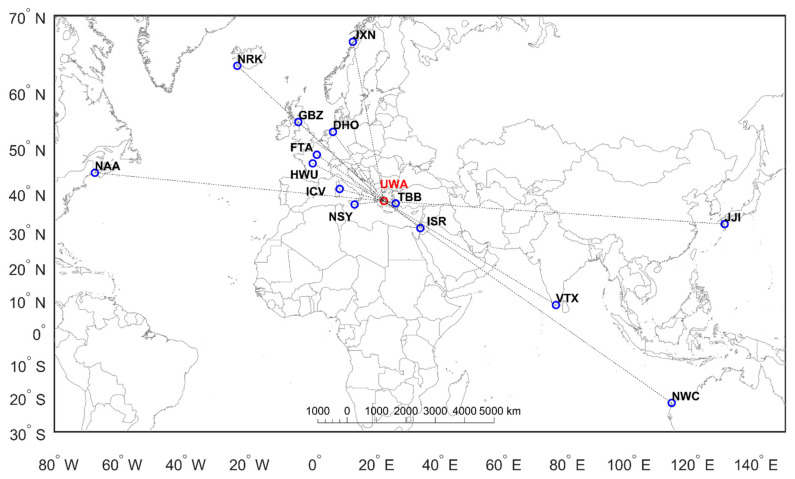
Map showing the locations along with the call signs of all of the transmitters worldwide that are recorded by the UWA receiver. The red circle denotes the location of the UWA receiver, whereas the blue circles indicate the locations of the monitored transmitters.

The UWA radio receiver records signals from several VLF transmitters worldwide, most of them located in Europe, whereas some of them are located in Asia, Australia, and North America (see Table 1 and Figure 1). It is noted that some of these VLF transmitters, which are mainly located in Europe, are also monitored by the INFREP (International Network for Frontier Research on Earthquake Precursors) network of VLF/LF receivers, also focusing on pre-seismic disturbances in the VLF/LF recordings [41,42].

It is worth mentioning that most of these monitored transmitters are operated for military purposes. Thus, there is little information about their technical characteristics, while their operation schedule is unknown. Consequently, it is difficult to anticipate their behavior, for the reason that they often change their operations suddenly without any notice. One of the problems in efficiently operating a VLF receiver is that the frequency of some transmitters is shifted regarding the last known value. In some cases, the shift is < 1 Hz, even of the order of mHz, leading to phase drifting with time. Hence, in such cases, one must proceed to transmitter frequency adjustment (fine tuning) by trial and error, until the observed diurnal variation of the phase is stabilized. Currently, we are still performing optimization tasks to our receiver concerning the monitoring of the transmitters of Table 1 (see also: Figure 1), and still evaluating the reception. Thus, UWA is still operating in trial mode, and the monitored transmitters may change in the future.

## 3. Earthquake and VLF Sub-Ionospheric Propagation Data

**Figure 2:**
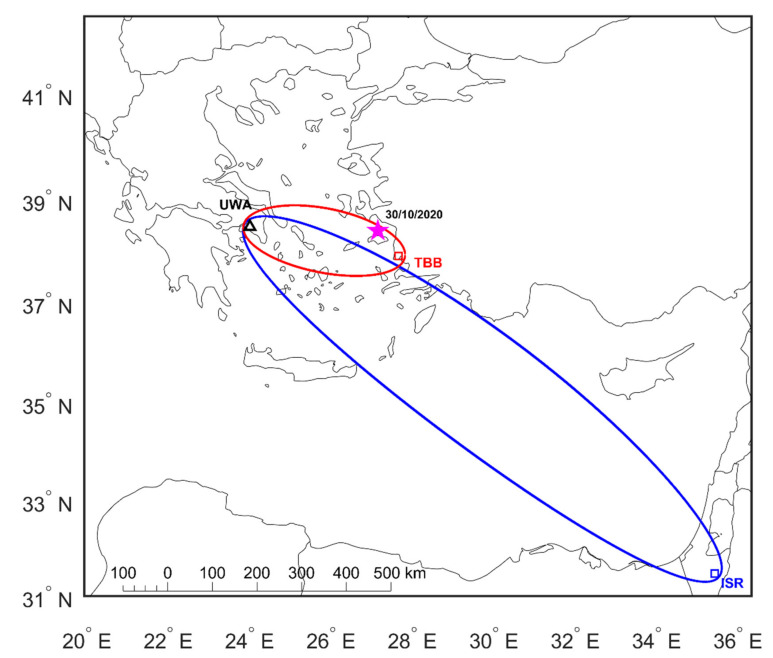
Map of the wider area of the Eastern Mediterranean. The transmitters TBB and ISR are indicated as red and blue rectangles, respectively, whereas the receiver UWA is indicated as a black triangle. The 5th Fresnel zones of the ISR–UWA and TBB–UWA VLF propagation paths are also shown on the map. The 2020 Samos EQ’s epicenter is indicated as a magenta star.

As already mentioned in Section 1, in this article we investigate the 2020 Samos EQ, a very recent, strong, and shallow EQ ($M_{W}=6.9$, focal depth = 12 km) that occurred on 30 October 2020 (time of occurrence 11:51:57 UTC) in Greece, with its epicenter located (geographic coordinates: 37.900° N, 26.816° E) in the Aegean Sea, off the coast of the island of Samos, close to the Greece–Turkey border (see Figure 2).

It has been statistically found that the lower ionosphere is sensitive to strong (M≥5.5) EQs when the epicenter of the EQ of interest is within or close to the 5th Fresnel zone of the considered propagation path [1,6,34]. It thus follows, among the VLF sub-ionospheric paths monitored by UWA receiver, that the TBB–UWA and ISR–UWA paths (see Figure 2 and Section 2) would be expected to have been influenced by the EQ preparation processes that resulted in the 2020 Samos EQ. Specifically, as shown in Figure 2, the epicenter of the 2020 Samos EQ is within the 5th Fresnel zone of the TBB–UWA sub-ionospheric propagation path, and close to the border of the 5th Fresnel zone of the ISR–UWA path. Taking into account the magnitude of the specific EQ, we consider that the ISR–UWA propagation path could possibly be disturbed, although the EQ epicenter is not within the corresponding 5th Fresnel zone.

In this study we analyze the VLF data of the above-mentioned paths for the time period from 1 October 2020 to 8 November 2020, i.e., from 1 month before to ~1 week after the 2020 Samos EQ. It has to be mentioned that no other EQs of M≥5.5 occurred within or close to the 5th Fresnel zones of the considered paths during this specific time period, also taking into account the aftershock sequence of the 2020 Samos EQ (see also: Section 3).

At this point, it has to be mentioned that the ionosphere is known to be sensitive not only to EQ preparation processes, but also to a variety of different phenomena such as solar flares, magnetic storms, volcanic eruptions, tsunamis, and typhoons [33,34,43]. We verified that there were not any other ionosphere-influencing phenomena or EQs during the analyzed time period in the wider area of interest, and for this reason our results are considered to be related to the 2020 Samos EQ alone. Specifically, we checked the EQ catalogues provided by the U.S. Geological Survey (https://earthquake.usgs.gov, accessed on 25 May 2021), the European–Mediterranean Seismological Centre (https://www.emsc-csem.org/Earthquake/, accessed on 25 May 2021), and the Institute of Geodynamics of the National Observatory of Athens (http://www.gein.noa.gr/en/seismicity/maps, accessed on 25 May 2021) for the time period 1 October 2020–30 November 2020, and no EQ with magnitude > 5.5 occurred within or close to the 5th Fresnel zones of the ISR–UWA and TBB–UWA VLF propagation paths. Moreover, we checked the disturbance storm time (Dst) index provided by the Data Analysis Center for Geomagnetism and Space Magnetism of Kyoto University (http://wdc.kugi.kyoto-u.ac.jp, accessed on 25 May 2021), and found that for the time period 1 October 2020–30 November 2020, all Dst index values were >−50 nT, indicating that no magnetic storms took place during the specific time period. Additionally, no solar flares of the M or X class occurred during the aforementioned time period, as evident from the catalogue provided by the Hinode Science Center at Nagoya (https://hinode.isee.nagoya-u.ac.jp/flare_catalogue/index.html, accessed on 25 May 2021). Finally, no volcanic eruptions happened in the area and time period according to the Global Volcanism Program of the Smithsonian Institution (https://volcano.si.edu, accessed on 25 May 2021), while no tsunami or typhoon was recorded before the 2020 Samos EQ.

In our investigation for any pre-seismic signatures in the lower ionosphere prior to the 2020 Samos EQ, we used only the amplitude data of the two aforementioned paths (TBB–UWA and ISR–UWA), because the phase data were not adequately stable during the studied time period. It is also noted that the first transmitter, with the call sign TBB, is located in Denizköy (Turkey), which is very close (~328 km) to our receiver; thus, the ground wave is considered to be stronger than the sky wave. The other transmitter, with the call sign ISR, is located in the Negev (Israel), which is close (~1304 km) to our receiver, and thus the ISR–UWA propagation path is characterized as a relatively short one.

## 4. Statistical and Criticality Analysis Methods

In this section we briefly present key information about both the statistical and the criticality time series analysis methods used for the analysis of the VLF data of the two considered sub-ionospheric propagation paths (TBB–UWA and ISR–UWA) in regard to the 2020 Samos EQ. Specifically, in Section 4.1 we present the nighttime fluctuation method (NFM), in Section 4.2 we present the terminator time method (TTM), while in Section 4.3 and Section 4.4 we present the natural time (NT) analysis method and the method of critical fluctuations (MCF), respectively.

### 4.1. Nighttime Fluctuation Method (NFM)

One of the most widespread statistical methods for the analysis of VLF data is the NFM [35], which can be summarized in three main steps. Firstly, we use the raw amplitude data (in dB) and calculate the residual (nighttime) amplitude signal $dA\left(t\right)$, by subtracting the average nighttime amplitude $A\left(t\right)$ of 31 days (±15 days around the day of interest plus the day of interest) from the nighttime amplitude $A\left(t\right)$, i.e., $dA\left(t\right)=A\left(t\right)-A\left(t\right)$. We must mention here that in this paper we use a specific nighttime interval: 18:00–02:00 UT (20:00–04:00 LT Greece). Secondly, we calculate daily values (one value per day) for three VLF propagation quantities TR (“trend”), DP (“dispersion”), and NF (“nighttime fluctuation”), as:

(1) $$\mathrm{TR}\;=\;\frac{\sum_{N_{s}}^{N_{e}}dA\left(t\right)}{N_{e}-N_{s}}$$

where TR represents the mean value of $dA\left(t\right)$*,* and $N_{e}$, $N_{s}$ are the ends of the chosen nighttime interval (starting and ending time points);

(2) $$\mathrm{DP}\;=\;\sqrt{\frac{1}{N_{e}\;-\;N_{s}\;}\overset{N_{e}}{\underset{N_{s}}{\sum }}{\left(dA\left(t\right)-TR\right)}^{2}}$$

where the DP is actually the standard deviation of $dA\left(t\right)$, and:

(3) $$\mathrm{NF}\;=\;\overset{Ne}{\underset{N_{s}}{\sum }}{\left(dA\left(t\right)\right)}^{2}$$

i.e., the NF denotes the power of $dA\left(t\right)$.

Thirdly, at this point, after the construction of the daily valued time series of the three above-defined VLF propagation quantities, normalization is applied for each of them by calculating the new daily valued time series $TR^{*}$, $DP^{*}$, and $NF^{*}$ as $X^{*}=\left(X-X_{\pm 15}\right)/\sigma_{\pm 15}$, where $X_{\pm 15}$ and $\sigma_{\pm 15}$ are the mean value and the standard deviation of ±15 days around the day of interest, respectively. Any statistical anomaly in these normalized time series exceeding ±2σ is investigated as possibly being EQ-related. We should also note that the trend and dispersion VLF propagation quantities are generally independent of one another (trend as the primary importance), while the nighttime fluctuation quantity is the combination of the other two quantities (trend and dispersion). Finally, we have to clarify that the above-presented means of applying the NFM, using a running window of ±15 days around the day of interest, is the standard means of applying the NFM for a posteriori statistical analysis that appears in the literature, e.g., [11,25,35,38]. This method of application clearly uses “future” information in the calculation of statistical quantities. Thus, if one is to apply the NFM in real time, or within the frame of an EQ forecasting system, 30 days before the day of interest plus the day of interest should be used for the calculation of statistical quantities.

### 4.2. Terminator Time Method (TTM)

The TTM is a very popular statistical method for the analysis of VLF data, and has been applied to several studies in the past [34,35,38,44,45]. This method emphasizes the occurrence time of the minima of the VLF signals (amplitude and phase) that appear close to the local (planetary) sunrise time and sunset time. These minima are referred to as sunrise terminators (SRTs) and sunset terminators (SSTs), respectively, or generally as terminator times (TTs), and are created from the interference of different propagation waves (modes of propagation) of the VLF signal—that is, the ground wave and the sky wave [37]. A significant shift in the SRTs or SSTs, as compared with neighboring days, is considered to be an anomaly before an EQ, when the lower ionospheric height is normally decreased [46]. In other words, an earlier appearance of an SRT or a late appearance of an SST, which means an anomalous increase of the duration of the “VLF day” (“VLF daylength”, $D_{VLF}$) as compared with the previous days, is considered to be an EQ precursor [37].

The TTM was initially applied to the strong Kobe EQ (M7.1) that occurred in Japan on 17 January 1995, for which significant shifts in the TTs appeared before the EQ’s occurrence [37,39]. Additionally, studies by Indian scientists have also reported shifts in TTs, and consequent increases in $D_{VLF}$, before an impending EQ [47,48,49,50]. Many other statistical studies have also reported correlations between EQs and TT anomalies, with maximal shifts occurring 0–4 days prior to the main EQ event [47,51,52,53,54,55,56,57,58]. Furthermore, some studies based on numerical simulation of the diurnal variation of the amplitude of the VLF signal, taking into consideration the characteristics of the VLF propagation path, the transmitter, and the receiver, are applied for the determination of TTs [50,59].

In applying the TTM, we initially find the time of appearance of two minima in the diurnal variation of the signal (amplitude or phase), which are close in time with the planetary sunrise and sunset time of each day, respectively. Using these time locations, which are the morning and evening TTs, we form two TT time series—one for the morning minima, denoted as $t_{m}$, and one for the evening minima, denoted as $t_{e}$. Subsequently, we use ±2 days around the day of interest window (5 days width) to calculate the running mean for each of the aforementioned time series, forming 2 new time series designated as $t_{m}$ and $t_{e}$, for the morning and evening TTs, respectively. Finally, the running mean time series are subtracted from the respective TT time series to form the residual TT time series $dt_{m}=t_{m}-t_{m}$ and $dt_{e}=t_{e}-t_{e}$, respectively [37,39]. Moreover, we calculate the “VLF daylength” as $D_{VLF}=t_{e}-t_{m}$, and similarly to the TT time series, we consecutively calculate the running mean time series $D_{VLF}$ and the residual “VLF daylength” time series ${\mathrm{dD}}_{VLF}=D_{VLF}-D_{VLF}$. Any statistical anomaly in the residual TTs or the residual “VLF daylength” exceeding ±2σ of the whole considered time period is investigated as possibly being EQ-related. We must note that the specific procedure uses the running mean values in order to reveal the shift of the TTs or of the $D_{VLF}$ prior to an impending EQ by removing their seasonal variability.

### 4.3. Natural Time (NT) Analysis Method

The NT time series analysis method has initially been applied to ultra-low-frequency (≤1 Hz) seismic electric signals (SES) [60,61,62], and has been shown to be optimal for enhancing the signals in the time–frequency space [63]. The application of NT analysis to various seismo-EM signals, including VLF sub-ionospheric propagation data, has been presented in detail in [9]. In the following section we will briefly present the key notions of this method.

Initially, for a number of N events, we determine the NT of the occurrence of the k-th event as $x_{k}=k/N$. Next, we determine the “energy” of each event in NT, which is symbolized as $Q_{k}$ for the k-th event. At this point, we must mention that $Q_{k}$ corresponds to different kinds of quantities, depending on the time series under analysis. For example, in the case of seismic events, $Q_{k}$ is the seismic energy released (seismic moment), while for dichotomous SES signals, $Q_{k}$ corresponds to the SES pulse duration [61]. However, in the case of fracto-EM emission signals in the MHz band, which are non-dichotomous signals, $Q_{k}$ denotes the energy of each event by using consecutive amplitude values above a noise threshold, as described in [21].

Next, we study the evolution of the pair of $\left(\chi_{k},Q_{k}\right)$. The approach of a dynamical system to criticality is identified by means of the variance $\kappa_{1}=\langle x^{2}\rangle -{\langle x\rangle }^{2}$ of NT weighed with $p_{k}$, where pk=Qk∑n=1NQn is the normalized energy released during the k-th event and $\langle f\left(x\right)\rangle =\overset{N}{\underset{n=1}{\sum }}p_{k}f\left(x_{k}\right).$ Hence, the quantity $\kappa_{1}\;$can be written as $\kappa_{1}\;=\;\sum_{k=1}^{N+1}p_{k}\chi_{k}^{2}-{\left(\sum_{k=1}^{N+1}p_{k}\chi_{\kappa }\right)}^{2}$. Moreover, the entropy $\left(S_{nt}\right)$ in NT is defined as $S_{nt}=\sum_{k=1}^{N}p_{k}\chi_{k}\ln\chi_{k}-\left(\sum_{k=1}^{N}p_{k}\chi_{\kappa }\right)\ln\left(\sum_{k=1}^{N}p_{k}\chi_{k}\right)$, which corresponds to the value for q=1 of the derivative of the fluctuation function with respect to q, $\mathrm{fl}\left(q\right)$ (while $\kappa_{1}$ corresponds to $\mathrm{fl}\left(2\right)$) [10,64]. The entropy in NT is a dynamic entropy, depending on the order of the events [64]. Moreover, $S_{nt-}$, the entropy under time reversal $\left({\mathrm{Tp}}_{m}=p_{N-m+1}\right)$, is also studied [64].

In many studies on dynamical systems, it has been found that the value of $\kappa_{1}$ is a measure to quantify the extent of the organization of the system at the onset of the critical stage [10]. The criticality is reached when (a) $\kappa_{1}$ takes the value $\kappa_{1}=0.07$, and (b) both the entropy in NT and the entropy under time reversal simultaneously satisfy the condition $S_{nt},S_{nt-}<S_{u}=\;\left(\ln2/2\right)-1/4$ [10,65], where $S_{u}$ is the entropy of the uniform distribution in NT [10,64].

In the special case of NT analysis of foreshock seismicity [61,62,63,64,66], we study the evolution of the quantities $\kappa_{1,\;\;}\;S_{nt,\;\;}\;S_{nt-}$, and 〈D〉 over time, where 〈D〉 is the “average” distance between the normalized power spectra$\;\Pi \left(\tilde{\omega }\right)={\left|\sum_{k=1}^{N}p_{k}exp\left(j{\tilde{\omega }\chi }_{\kappa }\right)\right|}^{2}$, ($\tilde{\omega }$ stands for the angular frequency in NT) of the evolving seismicity, and the theoretical estimation of $\;\Pi \left(\tilde{\omega }\right)$ for $\kappa_{1}=0.07$, $\;\Pi_{\mathrm{critical}}\left(\tilde{\omega }\right)\approx \;1-\kappa_{1}{\tilde{\omega }}^{2}$. Moreover, an “event” for the NT analysis of seismicity is considered to be any data point (EQ) of the original seismicity time series that surpasses a magnitude threshold, $M_{Thres}$.

The analysis starts with an appropriate low threshold, and taking into account only an adequate number of events, which are first in the order of occurrence. Next, the subsequent events, in their original order, are one-by-one taken into account. For each additional event that is taken into account, the quantity $\chi_{k}$ is rescaled within the interval (0,1], while the normalized energy $p_{k}$ and the values $\kappa_{1,\;\;}\;S_{nt,\;\;}\;S_{nt-}$, and 〈D〉 are all re-calculated. In this way, a temporal evolution of these quantities is obtained, taking into account the current event and all preceding events. The described procedure is repeated for several, increasing, values of $M_{Thres}$ for each studied geographic area, and everything is repeated for different overlapping areas.

The seismicity is considered to be in a true critical state, and a “true coincidence” is achieved, as soon as (a) $\kappa_{1}$ takes the value $\kappa_{1}=0.07$, (b) both the entropy in NT and the entropy under time reversal simultaneously satisfy the condition $S_{nt},S_{nt-}<S_{u}$, and three additional conditions are satisfied: (c) The “average” distance 〈D〉 should be smaller than $10^{-2}$, i.e., $\langle D\rangle =\langle \left|\Pi \left(\tilde{\omega }\right)-\Pi_{critical}\left(\tilde{\omega }\right)\right|\rangle <10^{-2}$ (this is a practical criterion for signaling the achievement of spectral coincidence) [10]; (d) the parameter $\kappa_{1}$ should approach the value $\kappa_{1}=0.070$ “by descending from above”, i.e., before the main event the parameter $\kappa_{1}$ should gradually decrease until it reaches the critical value 0.070 (this rule was found empirically) [10,62]; and (e) the above-mentioned conditions (a–d) should continue to be satisfied even if the considered $M_{Thres}$ or the area within which the seismicity is studied are changed (within reasonable limits).

The use of the magnitude threshold excludes some of the weaker EQ events (those events whose magnitude is <$\;M_{Thres}$) from the NT analysis. However, the usage of the magnitude threshold is valid for the reason that some recorded magnitudes are not considered reliable due to the seismographic network. On the other hand, the application of various $M_{Thres}$ values is useful in determining the time range within which criticality is reached. This is because, in some cases, it is found that multiple time points may satisfy the rest of the NT critical state conditions (a–d), and criterion (e) is the one that finally reveals the true time of criticality.

For the application of NT analysis to VLF data, we follow the paradigm of the NT analysis of seismicity, by using the non-normalized VLF propagation quantities (defined in Section 4.1) to define the “energy” $Q_{k}$ and the necessary threshold values as in [25] (see Section 5.3).

### 4.4. Method of Critical Fluctuations (MCF)

It has been proposed that EQ-related phenomena can be studied from the point of view of phase transition phenomena [67], characterized by the transition between two phases (states) in which a system could exist. That is, as the Earth’s crust system evolves towards a specific main EQ event, it experiences different states [29,68,69,70]. The MCF is a time series analysis method that is able to monitor the dynamics of the order parameter fluctuations; namely, the critical dynamics, and the departure from the critical state, either by the emergence of tricritical dynamics or by appearance of the so-called “spontaneous symmetry breaking” (SSB) phenomenon [9,70,71]. The MCF has been applied to a variety of time series which correspond to different observables of complex systems in many scientific fields, including the geophysical, biological, economic, thermal, and electronic sciences [17,18,19,20,22,23,24,27,28,72,73,74,75,76,77]. The application of the MCF to various seismo-EM signals, including VLF sub-ionospheric propagation data, has been presented in detail in [9]. In the case of VLF sub-ionospheric propagation data, the MCF is applied to the raw linear amplitude data (restored from the originally recorded dB values). In the following section we will briefly present the key notions of this method.

It has been shown that the dynamics of the fluctuations of the order parameter at the critical state can be modeled by the one-dimensional nonlinear intermittent map [14,16]:

(4) $$\phi_{n+1}=\phi_{n}+u\phi_{n}^{z}+\varepsilon_{n}$$

where $\phi_{n}$ is the n-th sample of the scaled order parameter, z is a characteristic exponent, and u>0 is a coupling parameter. The shift parameter $\varepsilon_{n}\;$represents the non-universal uncorrelated noise. Moreover, it is mentioned that the exponent z for a thermal system is associated with the isothermal critical exponent δ as z=δ+1.

In the critical state, the plateau region of the invariant density $P\left(\phi \right)$ corresponds to the laminar region of the critical map, where fully correlated dynamics take place. The start of the laminar region is the fixed point (f.p.) $\phi_{0}$, determined by the edge of the most “abrupt” side of $P\left(\phi \right)$, while the end of the laminar region $\phi_{L}$ is not exactly defined [9]. Consequently, the parameter $\phi_{L}$ should be used as a varying parameter in the application of the MCF.

An important observation in the application of the MCF is the fact that the distribution $P\left(L\right)$ of the laminar lengths L (i.e., of the time intervals for which ϕ stays within the considered laminar region) of a time series produced by the map of Equation (4) in the limit $\varepsilon_{n}\to 0$ is given by the power-law relation [78]:

(5) $$P\left(L\right)\;\sim \;L^{-p_{L}}$$

Thus, the exponent $p_{L}$ is pL=zz−1, and is connected to the isothermal exponent δ by pL=1+1δ. This power-law relation is related to the aforementioned plateau of the invariant density $Ρ\left(\phi \right),$ and is a signature of the underlying correlated dynamics related to critical behavior [18].

In detecting the critical state, the MCF is focused on revealing such power laws and estimating the exponent $p_{L}$. For this purpose, a truncated power-law function $f\left(L\right)$ is used to model the $P\left(L\right)$ resulting from each considered $\phi_{L}$:

(6) $$f\left(L\right)\;=\;p_{1}\;L^{-p_{2}}\;e^{-Lp_{3}}.$$

If $p_{3}=0$ is zero, then $p_{2}$ is equal to the exponent $p_{L}$ of Equation (5). Since, according to the theory of critical phenomena, the isothermal critical exponent δ is higher than 1 [79], and, as already mentioned, z=δ+1, pL=zz−1 for the critical state holds that $1<p_{L}\;\left(\;=\;p_{2}\right)<2$. Therefore, the critical state calls for the satisfaction of the conditions $p_{2}>1$ and $p_{3}\approx 0$.

As already mentioned, the departure from the critical state is signified either by the emergence of tricritical dynamics or by appearance of SSB. However, by means of the study of fracture-induced EM emissions in the MHz band in analogy to thermal systems, it has most recently been found that post-SSB (and post-EQ) power laws can be identified without being related to the preparation of a second main EQ [71]. Specifically, in a possible identification of post-SSB power laws immediately after a very strong EQ, if the values of the autocorrelation function of the examined time series collapse immediately after the EQ and remain low, then no new strong EQ is expected, but if the autocorrelation function values return to high values, then a new strong EQ may be expected soon [71]. In the first case, the post-EQ power laws in the distribution of laminar lengths, which are not accompanied by long memory in the corresponding autocorrelation function, are not related to mainshock preparation processes, but are attributed to local fractures in course of the aftershock sequence, which are not able to organize the system towards the preparation of a new mainshock [71].

## 5. Analysis of the Lower Ionosphere Prior to the Samos EQ

In this section we present the analysis results of the UWA VLF/LF receiver amplitude data for a wide time period, from 1 October 2020 to 8 November 2020, including almost 1 week after the date of the EQ, for the sub-ionospheric propagation paths TBB–UWA and ISR–UWA, by means of statistical and criticality analysis methods (see Section 4). This section comprises five subsections. Specifically, in Section 5.1 we present the results of the conventional NFM analysis (see Section 4.1); in Section 5.2 we present sequential plots of the daily variation of the raw amplitude data, where anomalies in the evolution of TTs are identified by inspection of the diurnal variation, while we also present the shift of TTs by applying the TTM analysis (see Section 4.2); in Section 5.3 we present the results of the NT analysis (see Section 4.3) as applied to the time series of the three non-normalized VLF propagation quantities (TR, DP, and NF) of the NFM; in Section 5.4 we present the results of the MCF analysis (see Section 4.4) as applied to the raw linear amplitude data; in Section 5.5 we summarize and discuss all of our findings.

### 5.1. NFM Analysis Results

**Figure 3:**
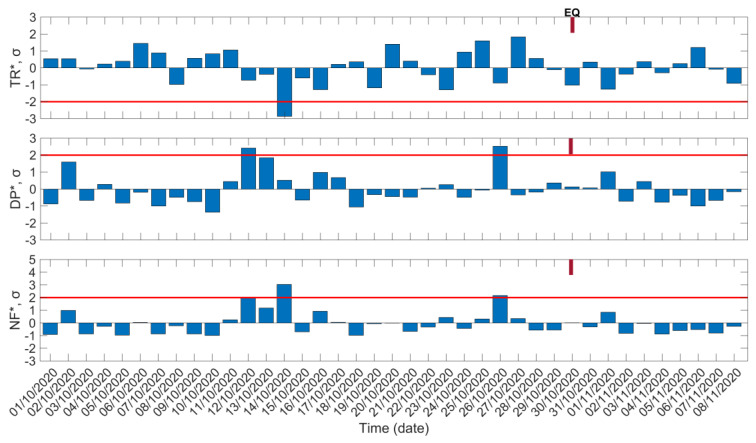
Temporal evolution of the three normalized VLF propagation quantities of the NFM for the TBB−UWA propagation path: $TR^{*}$ (top panel), $DP^{*}$ (middle panel), and $NF^{*}$ (bottom panel). Red solid horizontal lines indicate the corresponding −2σ/+2σ limits; σ is calculated for the whole studied period for each panel. EQ date is marked on top of each panel.

In Figure 3, we present the results of the temporal evolution of the three normalized VLF propagation quantities $TR^{*}$ (“normalized trend”), $DP^{*}\;$(“normalized dispersion”), and $NF^{*}$ (“normalized nighttime fluctuation”) (see Section 4.1), respectively, during the time period 1 October 2020–8 November 2020, for the propagation path TBB–UWA (see Figure 2). The standard deviation, σ, of each analyzed time series has been calculated for the whole studied period. The corresponding −2σ or +2σ level, indicating the considered threshold for an anomaly, is also indicated in each panel. It should be mentioned that, in applying the NFM to TBB–UWA, on the dates 28 October 2020 and 29 October 2020 we have excluded two excerpts of sudden artificial disturbances from the nighttime fluctuations of the amplitude, when the TBB transmitter was out of order, maintaining only the natural fluctuations of the amplitude. In Figure 3 it is observed that on 14 October 2020, 16 days before the 2020 Samos EQ, a very clear depletion of $TR^{*}$, greatly exceeding the −2σ limit, is found, and correspondingly an enhancement of $NF^{*}$, exceeding the +2σ limit, is observed on the same day. On 12 October 2020, 18 days prior to the 2020 Samos EQ, a marginal enhancement of $DP^{*}$, as well as a clear enhancement of $NF^{*}$, were found. On the other hand, an enhancement of $DP^{*}$, exceeding the +2σ limit, can be seen on 26 October 2020, very close to the EQ, 4 days before the mainshock, while in parallel an enhancement of $NF^{*}$, above the +2σ limit, is also observed. Summarizing, two out of the three examined normalized VLF propagation quantities present anomalies for each of the dates 12 October 2020, 14 October 2020, and 26 October 2020. Therefore, for all three dates it is considered that the corresponding lower ionospheric anomalies were possible precursors to the 2020 Samos EQ, even though the anomalies of the dates 12 October 2020 and 14 October 2020 appeared more than 2 weeks before the EQ, which is the longest time distance of such anomalies from the EQ occurrence, based on results from similar studies concerning EQs in Japan [1].

**Figure 4:**
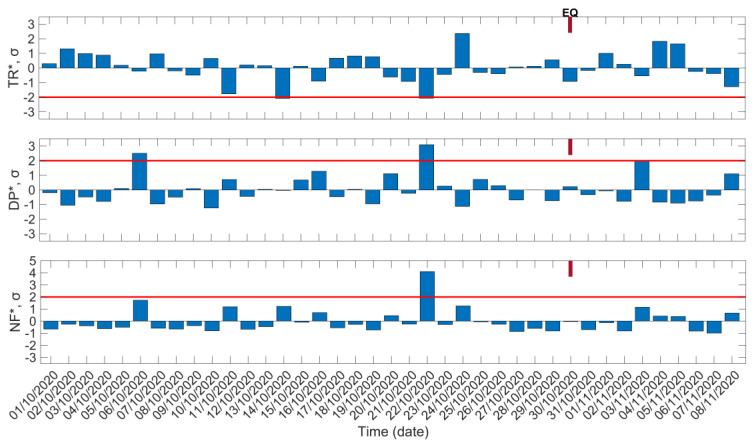
Temporal evolution of the three normalized VLF propagation quantities of the NFM for the ISR−UWA propagation path: $TR^{*}$ (top panel), $DP^{*}$ (middle panel), and $NF^{*}$ (bottom panel). Red solid horizontal lines indicate the corresponding −2σ/+2σ limits; σ is calculated for the whole studied period for each panel. EQ date is marked on top of each panel.

In Figure 4 we present the results of the three normalized VLF propagation quantities of NFM for the propagation path ISR–UWA for the same examined period (1 October 2020–8 November 2020). Firstly, we observe a depletion of $TR^{*}$ on 14 October 2020. Interestingly, as already mentioned, on the same date, simultaneous anomalies of $TR^{*}$ and $NF^{*}$ were already found for the propagation path TBB–UWA (Figure 3). The simultaneous appearance of these anomalies on 14 October 2020, almost 2 weeks prior to the 2020 Samos EQ, in both of the examined propagation paths is highly unlikely to be accidental, and is considered to be a significant precursor signature. On the other hand, on 14 October 2020 we do not observe any anomaly for $DP^{*}$ and $NF^{*}$ in Figure 4. Another anomaly is identified in Figure 4 on 6 October 2020 as an increase of $DP^{*}$ above the +2σ limit without any significant depletion in trend. This specific anomaly is not considered to be related to the 2020 Samos EQ, for the reason that only one normalized VLF propagation quantity presents the anomaly, and in addition the date of the anomaly is very far from the EQ’s occurrence. On the other hand, almost 1 week prior to the 2020 Samos EQ, on 22 October 2020, anomalies exceeding the corresponding −2σ/+2σ limits are simultaneously identified in Figure 4 for all three VLF propagation quantities ($TR^{*}$, $DP^{*}$, and $NF^{*}$), which are considered valid precursors to the EQ of interest.

### 5.2. Diurnal Variation and TTM Analysis Results

**Figure 5:**
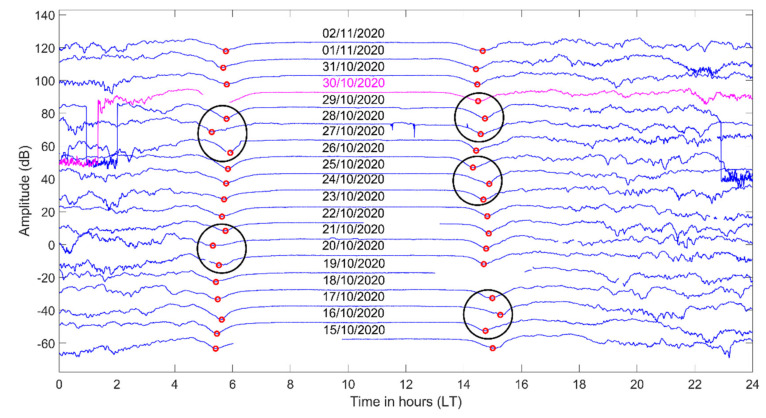
Diurnal variation of the amplitude of the VLF signal for the propagation path TBB−UWA for the time period 15 October 2020–2 November 2020. Each signal is vertically shifted by +10 dB in regards to the signal of the previous day. Red circles indicate the minima identified as the morning TTs $t_{m}$ (closest to the sunrise time), and the evening TTs $t_{e}$ (closest to sunset time), respectively. The EQ day is marked with magenta color. The anomalous shifts in TTs are marked with black ellipses.

In Figure 5, we present the sequential plot of the diurnal variation of the filtered (by a Gaussian low pass filter) amplitude data for the propagation path TBB–UWA. The time axis shows the local time (LT) at the location of the UWA receiver (EET = UT + 2 h), and the presented time period is 15 October 2020–2 November 2020. Note that the amplitude level (see level of the 15 October 2020 signal) received at UWA from the TBB transmitter during the considered time period was adequately high (>40 dB above noise level). The minima of the amplitude signal close to the local (planetary) sunrise and sunset times are identified as the morning TTs, $t_{m}$, and the evening TTs, $t_{e}$, respectively. We indicate these minima as red circles in Figure 5, while the dates are shown in the middle of each of diurnal variation of the signal, and the EQ day is marked with magenta color. The observed anomalous shifts in time of those minima, for different days prior to the 2020 Samos EQ, are marked with black ellipses, including the previous and the next normal days around the anomaly. It is noted that for a clearer presentation of the specific sequential plot, we intentionally removed specific excerpts of the signal for some days (see data gaps in Figure 5), because these corresponded to artificial disturbances due to transmitter operation disruptions and would “hide” some of the minima.

As regards the $t_{m}$, a significant reduction (shift towards the night) appears on 28 October 2020, 2 days prior to the 2020 Samos EQ, while a gradual increase of $t_{e}$ (towards the night) starts 4 days before the date of the EQ, maximizing on 29 October 2020, almost 1 day prior to the EQ. Moreover, $t_{e}$ presents another obvious anomaly on 25 October 2020. Note that the lead time of TT anomalies has been observed to be ~1 week prior to the EQ [37,39]. Therefore, the aforementioned anomalous shifts of the TTs could be considered to be possible precursors to the EQ. However, one anomalous shift of $t_{m}$ is identified on 21 October 2020, and one more is identified on 17 October 2020 for $t_{e}$; both of these anomalies exceed the lead time of ~1 week prior to the EQ.

**Figure 6:**
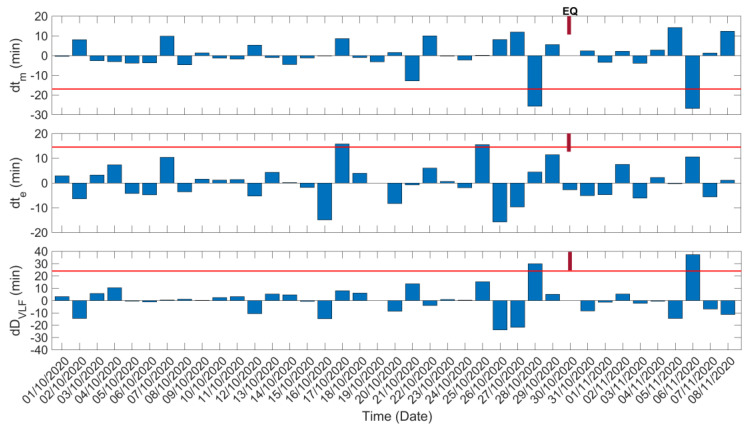
Temporal evolution of the shifts in the morning and evening TTs, as well as of the “VLF daylength” for the propagation path TBB−UWA during the studied period. Red solid horizontal lines indicate the corresponding −2σ/+2σ limits; σ is calculated for the whole studied period for each panel. EQ date is marked on top of each panel.

In Figure 6, we present the TTM analysis results for the TBB–UWA propagation path. In this figure we show the shift of the morning and evening TTs$—dt_{m}$ and $dt_{e}$, respectively—as well as the shift of the “VLF daylength”, ${\mathrm{dD}}_{VLF}$, as described in Section 4.2. The standard deviation, σ, of each analyzed time series has been calculated for the whole studied period. The corresponding −2σ or +2σ level, indicating the considered threshold for an anomaly, is also indicated in each panel.

Firstly, it is observed that on 28 October 2020 an anomalous shift of $t_{m}$ (decrease by −25.7 min), exceeding the −2σ limit, appears, while on the same date, $D_{VLF}$ also presents an anomalous increase by 29.9 min, exceeding the +2σ limit. Recall that an anomalous shift of the TTs has already been noticed on the specific date from the inspection of the sequential plot of the diurnal variation of the amplitude data (Figure 5). Thus, these TT anomalies could be characterized as a precursor to the studied EQ.

From Figure 6, one can also identify two more anomalies in the $dt_{e}$ time series, on 17 October 2020 (increase of $t_{e}$ by 15.79 min) and on 25 October 2020 (increase of $t_{e}$ by 15.48 min). These two TT anomalies are not considered to be clear precursors to the 2020 Samos EQ, for the reason that no corresponding anomalous increase of $D_{VLF}$ is observed on these dates. A decrease (by −26.78 min) of $t_{e}$, as well as an increase (by 37.32 min) of $D_{VLF}$, both exceeding the +2σ level, appear after the occurrence of the EQ (on 6 November 2020); however, these cannot be linked with any phenomenon.

**Figure 7:**
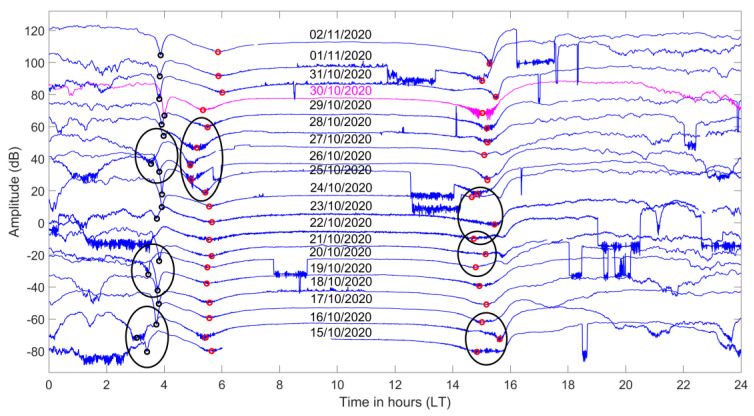
Diurnal variation of the amplitude of the VLF signal for the propagation path ISR−UWA for the time period 15 October 2020–2 November 2020. Each signal is vertically shifted by +10 dB in regards to the signal of the previous day. The first appearing minima (before sunrise), marked with black circles, are identified as the morning TTs $t_{m2}$, while red circles indicate the minima identified as the morning TTs $t_{m1}$ (closest to the sunrise time), and the evening TTs $t_{e}$ (closest to sunset time), respectively. The EQ date is marked with red font color. The anomalous shifts in TTs are marked with black ellipses.

Following the format of Figure 5, we present in Figure 7 the sequential plot of the diurnal variation of the filtered amplitude data for the propagation path ISR–UWA. As we can see from the Figure 7, the transmitter had a few interruptions in its operation during the time period presented. However, these interruptions do not influence the application of the TTM. Additionally, we observe that two sets of minima exist on the morning side; we chose to analyze both of them. The closest to the planetary sunrise time set of minima are designated as $t_{m1}$ (marked with red circles in Figure 7), whereas the set of minima appearing approximately 1 h earlier are denoted as $t_{m2}$ (marked with black circles in Figure 7). The evening TTs, $t_{e}$, appear also in Figure 7, as minima at the evening side, marked with red circles.

The observed TT shifts, indicated in Figure 7 as black ellipses that include the previous and the next normal days around the anomaly, appear at different days for each one of the three studied TTs. Specifically, one observes that three sequential $t_{m1}$ values are shifted towards the night on 26 October 2020, 27 October 2020, and 28 October 2020, close to the date of the EQ, while at the same time, on 27 October 2020, $t_{m2}$ is also shifted towards the night. These are considered to be significant anomalies, possibly related to the 2020 Samos EQ. Another two $t_{m2}$ anomalies are found in the morning side on 16 October 2020 and 20 October 2020, while for the evening TTs, $t_{e}$, we observe three distinct anomalies on 16 October 2020, 21 October 2020, and 23 October 2020. Note that these last anomalies appear more than 1 week prior to the 2020 Samos EQ.

**Figure 8:**
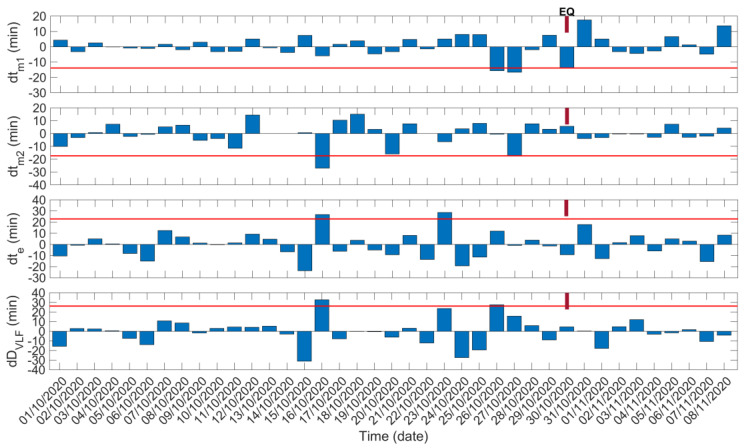
Temporal evolution of the shifts in the morning and evening TTs, as well as of the “VLF daylength” for the propagation path ISR−UWA during the studied period. Red solid horizontal lines indicate the corresponding −2σ/+2σ limits; σ is calculated for the whole studied period for each panel. EQ date is marked on top of each panel.

In Figure 8, following the format of Figure 6, we present the TTM analysis results for the ISR–UWA propagation path. The only difference is that in Figure 8 we present the results for two morning TTs, $t_{m1}$ and $t_{m2}$, instead of one. It is also noted that, for the ISR–UWA path, we calculated the “VLF daylength” as $D_{VLF}=t_{e}-t_{m1}$. Otherwise, the TTM has been applied to the ISR–UWA path exactly as it was applied to the TBB–UWA path.

From the results presented in Figure 8, we observe two anomalies of $t_{m1}$ close to the EQ occurrence, on 26 October 2020 ($t_{m1}$ shifted by −15.67 min) and on 27 October 2020 ($t_{m1}$ shifted by −16.63 min), as well as one anomaly of the ${\mathrm{dD}}_{VLF}$ time series on 26 October 2020 and one marginal exceed of the −2σ limit appearing for the $dt_{m2}$ time series on 27 October 2020. Recall that anomalous shifts of the $t_{m1}$ and $t_{m2}$ TTs have already been noticed on these specific dates by inspection of the sequential plot of the diurnal variation of the corresponding amplitude data (Figure 7). Thus, one could suggest that TTs indicate precursory behavior during the specific dates. Moreover, the $dt_{e}$ time series indicates an anomalous shift of $t_{e}$ on 23 October 2020, which also appears in Figure 7. Finally, on 16 October 2020, simultaneous anomalies of $t_{m2}$ (shifted by −26.96 min), $t_{e}$ (shifted by 26.86 min), and $D_{VLF}$ (shifted by 32.77 min) are found in Figure 8. Note that corresponding anomalous shifts of $t_{m2}$ and $t_{e}$ were already noticeable in Figure 7. Therefore, these anomalies are also considered to be significant precursors to the 2020 Samos EQ, although they appeared more than 1 week prior to the EQ.

### 5.3. NT Analysis Results

In this section we will give the results of the application of the NT method (see Section 4.3) to the time series produced by the NFM (see Section 4.3) by analyzing the non-normalized VLF propagation quantities TR, DP, and NF. We applied the NT method to these non-normalized VLF propagation quantities, as first appeared in [25]. Specifically, we consider all of the daily values of each one of the aforementioned VLF quantities that are higher than a certain threshold to be “events” to be taken into account during the NT analysis. The “energy” $Q_{k}$ of the k-th event is considered to be equal to the corresponding daily value of the analyzed VLF quantity, provided that this is above a certain threshold. Then, the NT analysis is applied to the time series of the events of each VLF quantity, as in the case of the pre-seismic activity (Section 4.3).

**Figure 9:**
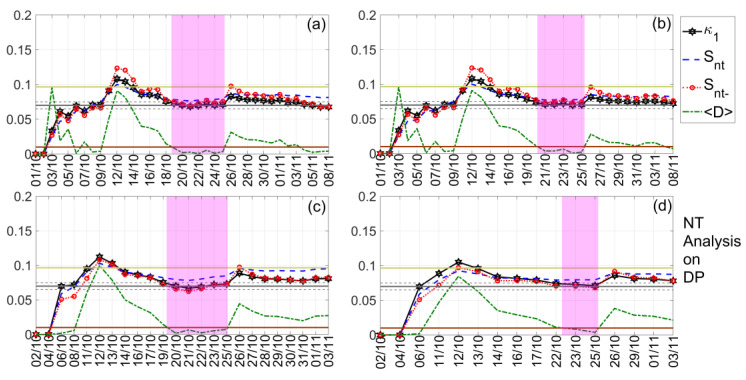
NT analysis of the DP VLF propagation quantity time series of the propagation path TBB–UWA for the examined time period (1 October 2020–8 November 2020). The presented temporal variations of the NT parameters correspond to the different thresholds of ${\mathrm{DP}}_{Th}$: (**a**) 0, (**b**) 0.3, (**c**) 1.8, and (**d**) 2.5, respectively. The limit value of the entropy $S_{u}\left(\approx 0.0966\right)$ appears as a horizontal solid light green line, while the $\kappa_{1}$ value 0.07, along with a region of ±0.05 around it, are denoted by a horizontal solid grey and two horizontal dashed grey lines, respectively. The $10^{-2}$ 〈D〉 limit is shown as a horizontal brown line. The events presented in each panel depend on the corresponding threshold. Moreover, although the conventional time (date) of occurrence of each corresponding event is noted in the x -axis tick values, the x -axis scale actually follows the NT representation; for this reason, the x− axis is not linear in conventional time. Date format is Day/Month (while for all dates the Year is the same: 2020).

**Figure 10:**
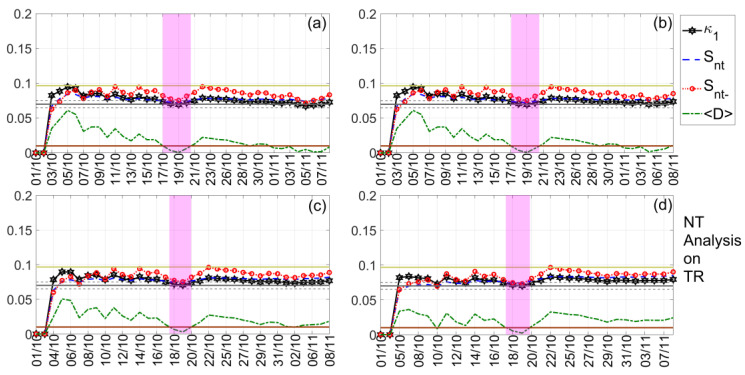
NT analysis of the TR VLF propagation quantity time series of the propagation path ISR–UWA for the examined period (1 October 2020–8 November 2020). The presented temporal variations of the NT parameters correspond to the different thresholds of ${\mathrm{TR}}_{Th}$: (**a**) 0.1, (**b**) 2.1, (**c**) 3.9, and (**d**) 5.3, respectively. Figure format follows the format of Figure 9.

**Figure 11:**
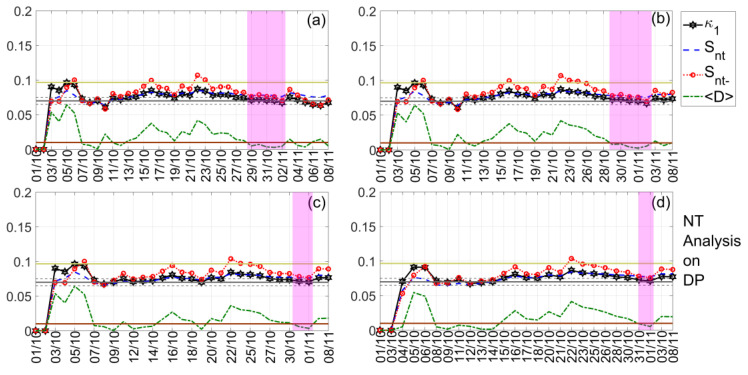
NT analysis of the DP VLF propagation quantity time series of the propagation path ISR–UWA for the examined period (1 October 2020–8 November 2020). The presented temporal variations of the NT parameters correspond to the different thresholds of ${\mathrm{DP}}_{Th}$: (**a**) 0, (**b**) 0.5, (**c**) 1, and (**d**) 1.3, respectively. Figure format follows the format of Figure 9.

In our investigation, we present the NT analysis results for the TR, DP, and NF time series of both TBB–UWA and ISR–UWA propagation paths, which are summarized in Table 2, while indicative results are shown in Figure 9, Figure 10 and Figure 11. Note that, although the NT analysis criticality conditions are satisfied for a number of thresholds for each case included in Table 2, in Figure 9, Figure 10 and Figure 11 only four indicative thresholds are presented per case.

In Figure 9, we present an example of the NT analysis of the DP VLF quantity of the TBB–UWA propagation path for the examined time period 1 October 2020–8 November 2020. It is shown that criticality is clearly achieved on 25 October 2020, since, on that date, for all 4 threshold values, ${\mathrm{DP}}_{Th}$, the $\kappa_{1}\;$ parameter is decreased to the 0.07 limit “from above”, while the entropies $S_{nt},S_{nt-}$ remain below the limit $\left(ln2/2\right)-1/4\;\left(\approx 0.0966\right)$, and the “average” distance D remains lower than $10^{-2}$. In Figure 10, we present another example concerning the NT analysis of the TR VLF quantity of the ISR–UWA propagation path for the same time period. It is found that criticality is reached on 19 October 2020, as the NT analysis criticality criteria are satisfied.

The last example is presented in Figure 11, where it is clearly observed that on 1 November 2020 (actually, 31 October–1 November 2020), notably after the occurrence of the main EQ, the system was still in critical condition. This criticality obviously cannot be related to the preparation of the 2020 Samos EQ, while no other phenomenon that could have disturbed the lower ionosphere occurred over a period of at least 1 month after this date (see also Section 3). Thus, this is a puzzling finding that calls for further investigation with the help of an independent criticality analysis method, such as the MCF. Moreover, it is questionable whether the criticality found on 30 October 2020 for the ISR–UWA path is related to the EQ preparation processes that resulted in the 2020 Samos EQ. Note that the NT analysis has been performed on daily valued time series, so that a criticality found on the day of the EQ may come from the part of the original VLF amplitude recordings preceding or the part following the EQ occurrence. Indeed, in Section 5.4., we proceed to such an investigation that explains these puzzling results.

### 5.4. MCF Analysis Results

In this section we present our MCF analysis results for the considered VLF data. We should clarify that the daily valued NFM VLF propagation quantities, which have been obtained by means of NT analysis, cannot be analyzed by means of the MCF for the reason that they comprise very few values, whereas the MCF needs approximately >5000 values [9] in order to produce reliable results. Here, the MCF is applied to the raw linear amplitude nighttime fluctuation data (restored from the originally recorded dB values) for the two propagation paths of interest (TBB–UWA and ISR–UWA), following the way in which the MCF was applied to VLF data in [24,30]. It should be mentioned at this point that in the case of the 2020 Samos EQ, there was no need to add uniform noise (see Section 4.4, and [9]), because the amplitude fluctuations for both propagation paths were sufficient for the application of the MCF. Thus, the MCF was directly applied to the stationary parts of the original time series, as was done in the time series of the fracture-induced EM emissions [8,23]. The time series excerpts presenting critical characteristics according to MCF are referred to as “critical windows” (CW).

It must be noted that CWs in seismo-EM signals usually appear up to 2 weeks before the mainshock; however, here we focus on the last few days before the 2020 Samos EQ, while we also examine the VLF data after the main EQ in order to investigate the puzzling finding of the NT analysis that criticality is also found on 1 November 2020, after the occurrence of the main EQ.

**Figure 12:**
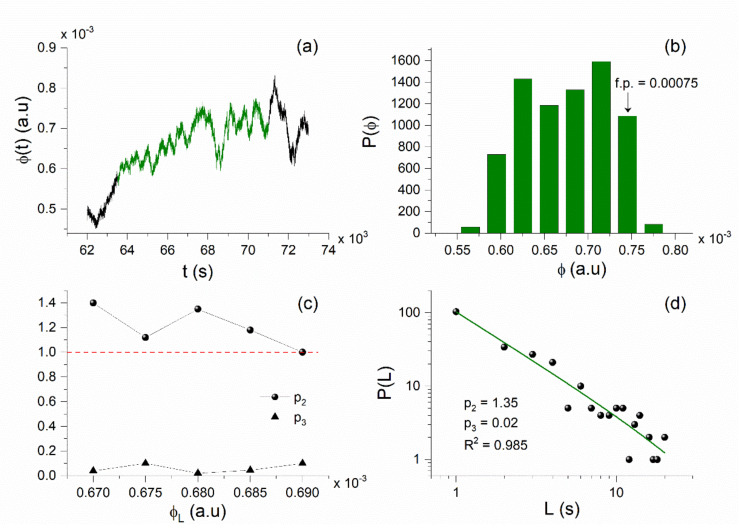
MCF analysis of a CW, identified in the VLF recordings of the ISR−UWA propagation path prior to the Samos EQ on 29 October 2020. (**a**) The green-colored excerpt is the analyzed excerpt of the VLF signal from 63,500 s to 71,000 s; time refers to the sample number within the specific day (sampling rate = 1 sample/s), starting at 00:00 UT. (**b**) The distribution of the green-colored excerpt of Figure 12a from which $\phi_{0}=0.00075$ is determined. (**c**) The estimated values for the exponents$\;p_{2}$, $p_{3}$ for different values of the end of the laminar region $\phi_{L}$. The red dashed horizontal line indicates the critical limit $\left(p_{2}=1\right)$. The criteria $p_{2}>1$ and $p_{3}\;≅0$ are satisfied for a wide range of $\phi_{L\;}$. (**d**) The distribution of the laminar lengths and the corresponding fitted power law (green solid line) for the laminar region determined by $\phi_{0}=0.00075$ and $\phi_{L}=0.00068$; $R^{2}\approx 1$ indicates an excellent fit.

**Figure 13:**
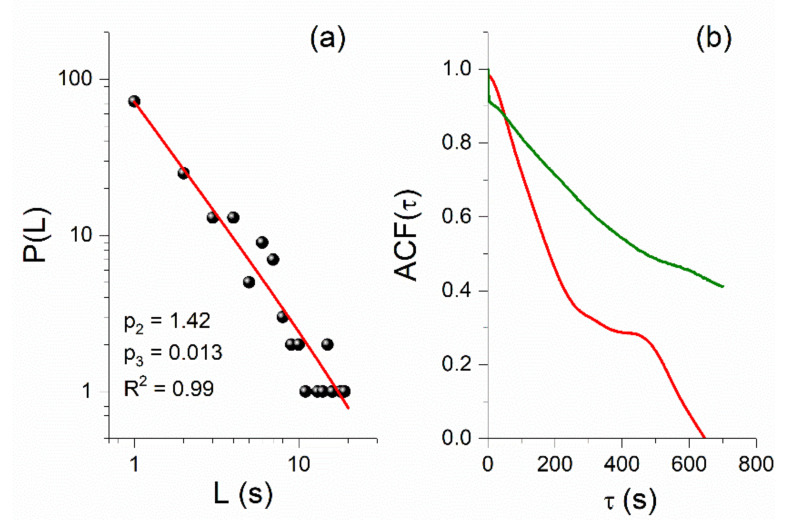
(**a**) Power-law behavior in the distribution of laminar lengths, resulting from the MCF analysis of an excerpt of the sub-ionospheric propagation amplitude time series for the ISR–UWA path, for the date 1 November 2020, (72,600 s–80,000 s, UT). (**b**) Autocorrelation function (ACF) of the time series excerpts corresponding to the power laws of Figure 12d (green curve), found ~1 day before the EQ, and Figure 13a (red curve), found ~2 days after the main EQ. It is clear that the post-EQ power law presents significantly shorter correlation length than the pre-EQ power law.

**Figure 14:**
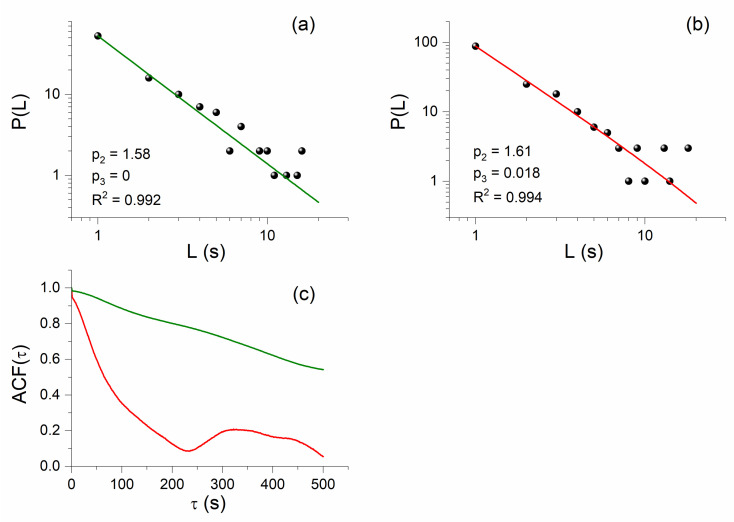
Power-law behavior in the distribution of laminar lengths, resulting from the MCF analysis of excerpts of the sub-ionospheric propagation amplitude time series for the TBB–UWA path, for the dates: (**a**) 29 October 2020 (56,000 s–61,500 s, UT), ~1 day before the EQ occurrence, and (**b**) 1 November 2020 (71,000 s–75,200 s, UT), ~2 days after the EQ occurrence. (**c**) Autocorrelation function (ACF) of the time series excerpts corresponding to the power laws of Figure 14a (green curve) and Figure 14b (red curve). It is clear that the post-EQ power law presents significantly shorter correlation length than the pre-EQ power law.

The analysis of both the ISR–UWA and TBB–UWA propagation paths in terms of the MCF revealed CWs before the 2020 Samos EQ, the last of which were found in the recordings of 29 October 2020 (see Figure 12, Figure 13a, and Figure 14a), i.e., 1 day prior to the mainshock, verifying the findings of the NT analysis for the existence of criticality before the mainshock in the analyzed VLF data.

In Figure 12, we show an example of the application of the MCF to one of the last in time CWs identified by the MCF, on 29 October 2020, in the VLF data of the ISR–UWA propagation path. Specifically, the amplitude fluctuation of part of the day (62,000 s–73,000 s, starting time at 00:00 UT) is illustrated in Figure 12a, while the excerpt presenting critical fluctuations, i.e., the CW, is marked with green color. The corresponding amplitude distribution of this excerpt is presented in Figure 12b, where the selected fixed point (f.p.), i.e., the $\phi_{0}$ value, for the MCF analysis is indicated (see Section 4.4). In Figure 12c, we give the sets of the exponents $p_{2},p_{3}$ for which the MCF criticality conditions $p_{2}>1$, $p_{3}\approx 0$ are satisfied. The intervals between $\phi_{0}$ and each one of the $\phi_{L}$ values define the corresponding laminar regions ($\phi_{0},\phi_{L}$)—that is, one laminar region ($\phi_{0},\phi_{L}$) for each different $\phi_{L}$ value. Finally, in Figure 12d, we present the distribution of laminar lengths (waiting times) within the laminar region determined by $\phi_{0}=0.00075$ and $\phi_{L}=0.00068$, as well as the corresponding fitting function $f\left(L\right)$ (see Section 4.4), which is a power law.

We now focus on the investigation of the puzzling finding of the NT analysis that criticality is also found on 30 October–1 November 2020. For this reason, we also analyzed the VLF data after the main EQ using the MCF. Indeed, we found power-law behavior in the distribution of laminar lengths of the sub-ionospheric propagation data for 1 November 2020, ~2 days after the 2020 Samos EQ, for both the ISR–UWA and TBB–UWA paths, as shown in Figure 13a and Figure 14b, respectively.

Since MCF criticality conditions $p_{2}>1$, $p_{3}\approx 0$ are satisfied for the specific cases (i.e., power-law behavior of the laminar lengths’ distribution), one could conclude that both of these post-EQ power laws indicate critical states and, therefore, that the corresponding time series excerpts are CWs. Such power laws, after the main EQ, were until recently attributed to the preparation of another, discrete, main EQ that may follow after the first one—or in the case of VLF data, to another extreme space-sourced geophysical phenomenon that is capable of disturbing the lower ionosphere. However, very recently, on the occasion of the analysis of the fracture-induced EM emissions in the MHz band that were recorded prior to the 2020 Samos EQ, it was found that such power-law findings in seismo-EM time series may not be related to the preparation of a new EQ [71]. Specifically, as already mentioned in Section 4.4, in such cases one must also check the autocorrelation function, $\mathrm{ACF}\left(\tau \right)$, of the corresponding time series excerpts. If the values of the autocorrelation function of the examined time series collapse immediately after the EQ and remain low, then no new strong EQ is expected, but if the autocorrelation function values return to high values, then a new strong EQ may be expected soon [71]. For both cases of post-EQ power laws presented in Figure 13a and Figure 14b, Figure 13b and Figure 14c clearly illustrate that the autocorrelation function values of the corresponding time series excerpts are significantly lower than those of the true critical windows that were identified before the 2020 Samos EQ (such as those corresponding to the power laws of Figure 12d and Figure 14a). It is worth noting that in [71] similar behavior was found for the fracture-induced EM emissions in the MHz band. Note also that all of the laminar distributions’ power laws found before the 2020 Samos EQ were accompanied by long-range correlations, as verified by means of the autocorrelation function of their corresponding time series excerpts.

As presented in detail in [71], by employing the 3D Ising thermal model, power laws in the laminar lengths’ distribution may appear after the SSB as a result of “locally surviving” critical dynamics, despite the unstable critical point no longer existing. The interpretation suggested in [71] for the post-EQ power laws is that they are due to local fracture structures (which explains the collapse of long-range correlations that are a key characteristic of the critical state), while critical dynamics “locally survive” due to the small temporal distance from the EQ occurrence. Such local fractures, in course of the aftershock sequence, due to the absence of long-range correlations, are not able to organize the system towards the preparation of a new mainshock.

At this point, it should be noted that due to the very low temporal resolution of the VLF propagation quantities of NFM that were analyzed by NT analysis (1 value per day), it was difficult for the NT analysis to discriminate between the criticality indications shortly before and shortly after the 2020 Samos EQ. For this reason, the post-EQ criticality found by the NT analysis in the DP VLF propagation quantity time series of the ISR–UWA path, as presented in Figure 11, seems to initially extend from 29 October 2020 to 1 November 2020, progressively focusing on 31 October–1 November 2020. For the same reason, the criticality found on 30 November 2020 for the NF VLF propagation quantity time series of the ISR–UWA path (Table 2) might well be related to the aforementioned critical dynamics “locally surviving” after the occurrence of the main EQ.

### 5.5. Summary of Analysis Results

Considering all of the results obtained by means of the statistical and criticality analyses applied to the VLF sub-ionospheric propagation data possibly disturbed by the 2020 Samos EQ (Section 5.1, Section 5.2, Section 5.3 and Section 5.4), and also taking into account the fact that no other extreme events (magnetic storm, solar flare, volcano, another EQ, etc.), which could affect the examined propagation paths occurred during the examined time period (1 October 2020–8 November 2020) or later (see also Section 3), one could summarize in the following comments:

1. Within ~1 week before the EQ, VLF anomalies have been revealed by means of the NFM statistical analysis method. Specifically, on 22 October 2020, the ISR–UWA path presents clear indications of disturbance in all the examined VLF propagation quantities, while on 26 October 2020 the TBB–UWA path also appears to be perturbed, since two out of the three examined VLF propagation quantities present anomalies.
2. Within ~2 weeks before the EQ, on 14 October 2020, both propagation paths present very clear anomalies, while on 12 October 2020 anomalies were found for 2 out of the 3 examined VLF propagation quantities for TBB–UWA path. These lower ionosphere perturbations are also considered to be related to the preparation of the 2020 Samos EQ, despite the fact that pre-seismic VLF anomalies usually appear up to ~1 week before the mainshock. In particular, the 14 October 2020 VLF anomalies that appear in both propagation paths are considered to be significant precursors to the EQ.
3. Both the earliest (12 October 2020) and latest (26 October 2020) anomalies appear in the TBB–UWA path.
4. The anomalies revealed for the TTs by inspection of the sequential plots of the diurnal variation of the VLF signal’s amplitude of both propagation paths imply that the lower ionosphere was disturbed within the time interval 25 October 2020–29 October 2020, while a TT anomaly was also found for the ISR–UWA path on 23 October 2020, all within ~1 week before the EQ in question. However, anomalies were also identified earlier—specifically, on 21 October 2020 and 17 October 2020 for the TBB–UWA path, as well as on 21 October 2020, 20 October 2020, and 16 October 2020 for the ISR–UWA path.
5. The application of the TMM statistical analysis indicate that the TTs’ anomalous behaviors on 28 October 2020 for the TBB–UWA path, as well as on 26 October 2020 and 27 October 2020 for the ISR–UWA path, are important precursory information, already observed by the inspection of the sequential plot of the diurnal variations. The same holds for the clear perturbation revealed by the TMM on 16 October 2020 for the ISR–UWA path, also observable in the corresponding sequential diurnal plot, although it appears ~2 weeks before the 2020 Samos EQ’s occurrence.
6. The criticality analysis performed by means of the NT analysis method provides evidence that the lower ionosphere was in the critical state within the time interval 17 October 2020–28 October 2020, within 2 weeks before the EQ of interest. Specifically, the TBB–UWA path first presents criticality from 17 October 2020 to 25 October 2020, while for the ISR–UWA path criticality appears between 19 October 2020 and 28 October 2020.
7. Moreover, criticality was also found by the NT analysis for the ISR–UWA path even on 1 November 2020, 1 day after the 2020 Samos EQ, but also on the day of the EQ. These puzzling findings were further investigated with the help of the MCF analysis.
8. The application of the MCF analysis verified that the lower ionosphere was in the critical state up to 1 day before the mainshock occurrence, while providing evidence that the criticality indications appearing after the occurrence of the 2020 Samos EQ lack long-range correlations and, thus, are attributable to critical dynamics “locally surviving” after the occurrence of the main EQ, and not to the preparation of any other extreme space-sourced geophysical phenomenon that could have disturbed the lower ionosphere.
9. The criticality analysis results of this study are extremely consistent with the criticality analysis results obtained by the used analysis methods on other EQ cases [9,24,25,30].

As already mentioned in Section 3, no other ionosphere-influencing phenomena occurred during the analyzed time period (or for at least 1 month after the EQ under study) in the wider area of interest. For this reason, the precursory evidence found is considered to be related only to the 2020 Samos EQ.

Finally, we must mention that if all of the NFM calculations are repeated using a running window of 30 days before the day of interest plus the day of interest for the calculation of statistical quantities, and these NFM results are used as inputs to the NT analysis, we will end up with the same conclusions summary for the NFM and NT analysis results.

## 6. Conclusions

This work has presented the statistical and criticality analysis of the VLF sub-ionospheric propagation data recorded by a newly installed radio receiver located in Athens (UWA), investigating the 2020 Samos EQ which occurred on 30 October 2020 in Greece. Specifically, we used the widespread statistical methods NFM and TTM, while for the criticality analysis we used NT analysis and the MCF. In the analysis we used the data of 2 sub-ionospheric propagation paths, corresponding to a transmitter located in Turkey (TBB) and one more transmitter located in Israel (ISR), for the time period 1 October 2020–8 November 2020, including almost 1 week after the EQ occurrence, knowing that no other extreme event that could affect the lower ionosphere occurred in the abovementioned period or for at least 1 month after the examined EQ’s occurrence.

Our findings indicate that precursory anomalies are identified in the lower ionosphere within a time interval of ~2 weeks prior to the 2020 Samos EQ. Since no other ionosphere-influencing phenomena occurred during the analyzed time period (or for at least 1 month after the EQ under study) in the wider area of interest, the precursory evidence found is considered to be definitely related to only the 2020 Samos EQ.

Interestingly, criticality indications were found even after the EQ’s occurrence, both by NT analysis and by the MCF. However, it was shown that these were not precursory signals of an EQ or other extreme event capable of influencing the lower ionosphere, but rather critical dynamics “locally surviving” after the occurrence of the main EQ, possibly due to local fracture structures, lacking long-range correlations, in course of the aftershock sequence, which were not able to organize the system towards the preparation of a new mainshock.

## Figures and Tables

**Table 1:** List of VLF/LF transmitters monitored by the UWA receiver.

No	Label	Country	Frequency (Hz)	Latitude	Longitude
1	DHO	Germany	23,400	53.0819° N	7.6163° E
2	GBZ	United Kingdom	19,580	54.9112° N	3.2813° W
3	JXN	Norway	16,400	66.9827° N	13.8731° E
4	FTA	France	20,900	48.5401° N	2.5502° E
5	HWU	France	21,750	46.7130° N	1.2444° E
6	ICV	Italy	20,270	40.9231° N	9.7310° E
7	NSY	Italy	45,900	37.1256° N	14.4363° E
8	TBB	Turkey	26,700	37.4094° N	27.3252° E
9	ISR	Israel	29,700	30.9756° N	35.0986° E
10	VTX	India	18,200	8.3870° N	77.7527° E
11	NWC	Australia	19,800	21.8161° S	114.1652° E
12	JJI	Japan	22,200	32.0453° N	130.8107° E
13	NRK	Iceland	37,500	63.8503° N	22.4664° W
14	NAA	United States	24,000	44.6463° N	67.2810° W

**Table 2:** NT analysis results for the propagation paths TBB–UWA and ISR–UWA. Only the transmitter data which presented clear satisfaction of the NT analysis criticality criteria are mentioned.

Date	TR	DP	NF
17 October 2020			TBB
19 October 2020	ISR		ISR
22 October 2020	TBB		
25 October 2020		TBB	
28 October 2020			ISR
30 October 2020			ISR
1 November 2020		ISR	

## Data Availability

Data are available upon request to the corresponding author.
